# Aconitia in Neuralgia

**Published:** 1880-06

**Authors:** 


### ARTICLE VIII.

### *Aconitia in Neuralgia.*

The affections known under the general name of neuralgia, which are so painful, and in the majority of cases so difficult to treat, have for a long time been the subject of constant investigation at the hands of a number of experimenters. Clinical experience has recently demonstrated the powerful anti-neuralgic action of crystallized aconitia, and the excellent results which have been obtained by the use of this remedy in the hands of Dr. Oulmont have fully confirmed the opinions in regard to it which have been already advanced. Aconitia, says Dr. Oulmont, is perfectly successful in such forms of facial neuralgia as are not correlated with other lesions which are not intermittent, and which have not a well marked recurrence; in other words, in those forms to which M. Gubler has applied the term congestive, and which are most frequently caused by exposure to cold. In such cases aconitia produces a rapid cure within two or three days. Dr. Oulmont has even seen a case of facial neuralgia of seven day's standing, in which there was no well marked periodicity, and which had resisted sulphate of quinia, yield instantaneously and permanently to a quarter of a milligramme of nitrate of aconitia (one milligramme is equal to one-sixtieth of a grain.) The results are more marked and rapid in cases of recent neuralgia than in those of long standing. Examples are quoted, however, in which the affection had lasted for periods of one month, two months, and even five years, but which had yet been cured, the first on the seventh day, the second on the third, and the last in three weeks. Aconitia has also a distinct effect in secondary neuralgia, as, for example, in dental caries, otitis, paraplegia, etc.

Acute rheumatic arthritis may be successfully treated with aconitia. In four individuals to whom this remedy was administered in doses, at first of half a milligramme per diem, increased gradually to one and a half milligrammes, a cure was affected, once in eight days, and once in ten days. The temperature fell from 39° to 36° (102 to 97° F.) and the pulse in proportion. In the other cases the cure was equally obtained, but only on the fifteenth and eighteenth days respectively, whilst the dose was raised to two and a half milligrammes. The antipyretic action, however, was equally well marked, whilst the temperature fell on the eighth and ninth days about two degrees. The results obtained by M. Gubler are also noteworthy. The results of four cases are published; in these the patients were treated with hypodermic injections of half a milligramme once or twice a day, whilst half a milligramme of aconitia, which was gradually increased till this quantity was taken two to four times a day, was administered internally. In these cases a cure was effected upon the sixth, ninth, twelfth, and thirteenth days; in one case there was a slight stiffness of the joints. The influence of the remedy upon the painful symptoms was very rapid upon the second to the fourth days, whilst upon the fever it was slower, though not less marked. The effects are very remarkable according to M. Gubler in cases of neuralgia of the fifth.

Dr. Oulmont concludes his work with the statement that aconitia is a remedy of importance, since it acts in a certain definite manner upon the human organism, but from its activity it must only be employed in very small doses and at long intervals. Neuralgia is often accompanied by intermittent symptoms and well marked periods. In such complications quinia must be employed in addition to aconitia. On account of the energetic action of the remedy the susceptibility of the patient should be tested by administering, in the first place, three pills daily, each containing a fifth of a milligramme of crystallized aconitia in addition to five centigrammes of pure quinia; one in the morning, one at midday, and one in the evening. If no alleviation of the pain is experienced on the first day, the dose may be cautiously augmented by a pill per diem, until a maximum dose of six in the course of twenty-four hours is attained, and in the majority of cases it will not be necessary to overstep this limit. If slight diarrhœa occurs, the dose must be reduced. Physiological experiments and clinical observations carried on in the Paris hospitals have shown us that these pills have a sedative influence upon the circulatory apparatus through the vaso-motor nerves, and it is concluded therefore that they are indicated in neuralgia of the fifth, in congestive neuralgia, in painful and inflammatory rheumatic affections, etc.—*Am. Jour. of Med. Sciences, from Le Progres Medical.*

